# Life-threatening diarrhea in neuroendocrine tumors: two case reports

**DOI:** 10.1186/s13256-021-03096-7

**Published:** 2021-10-27

**Authors:** Emma Gordon, David L. Chan, Jennifer Arena, Elizabeth Bernard, Emily Carr-Boyd, Stephen J. Clarke, Malinda Itchins, Diana Learoyd, Neomal Sandanayake, Nick Pavlakis

**Affiliations:** 1 Royal College of General Practitioners, 85 Tamar Street, Ballina, NSW 2478 Australia; 2 Department of Medical Oncology, Royal North Shore Hospital, St. Leonards, Sydney, NSW 2065 Australia; 3 Department of Nuclear Medicine, Royal North Shore Hospital (Sydney University), St. Leonards, Sydney, NSW 2065 Australia; 4 Department of Pathology, Royal North Shore Hospital, St. Leonards, Sydney, NSW 2065 Australia; 5 Genesis Care North Shore, North Shore Health Hub, Sydney, NSW 2065 Australia; 6 Department of Gastroenterology, Royal North Shore Hospital, St. Leonards, Sydney, NSW 2065 Australia

**Keywords:** Neuroendocrine tumor, Diarrhea, VIPoma, Case report

## Abstract

**Background:**

Neuroendocrine tumors are rare, heterogeneous neoplasms that produce a wide variety of clinical symptoms. Diarrhea in neuroendocrine tumors is incredibly common and is usually benign in nature. We report two extreme cases of diarrhea in metastatic neuroendocrine tumors that threatened fatality and provide evidence for steroids as a novel agent in the management of vasoactive intestinal peptide tumors.

**Case presentation:**

A 63-year-old Caucasian male with a grade 2 (Ki-67 17%) metastatic small bowel neuroendocrine tumor, and a 43-year-old female with a grade 2 (Ki-67 5%) metastatic pancreatic vasoactive intestinal peptide tumor. Both patients suffered life-threatening diarrhea despite extensive treatment modalities, including new systemic agents. This case explains how a lack of compliance and patient under-reporting of symptoms contributed to their challenging clinical course. Only steroids had a significant sustained effect on the diarrhea of the patient with vasoactive intestinal peptide tumor.

**Conclusions:**

This report discusses two rare cases of life-threatening diarrhea in neuroendocrine tumors and stresses the importance of accurate clinical history taking, patient education, and compliance for symptom control. The report suggests steroids as a potential novel pharmaceutical option in the management of vasoactive intestinal peptide tumors; this is of great significance as it may provide a new approach to their management and potentially act as a life-saving agent in other oncology patients.

## Background

Neuroendocrine tumors (NETs) are heterogeneous neoplasms that are classified according to their grade, the primary tumor site, and their ability to secrete vasoactive hormones. While “nonfunctional” NETs have no hormone-related clinical features, “functional” NETs have a highly variable clinical presentation based on the type and amount of hormone produced [1]. Approximately 20% of patients have carcinoid syndrome at the time of diagnosis with symptoms including diarrhea and flushing [2]. We present two extreme cases of life-threatening diarrhea in metastatic NETs to stress the importance of clinical history taking in these tumors and emphasize the impact symptoms can have on patients’ quality of life. The report offers a novel approach to management with the use of steroids in vasoactive intestinal peptide tumors (VIPomas).

## Case presentation 1

**Fig. 1:**
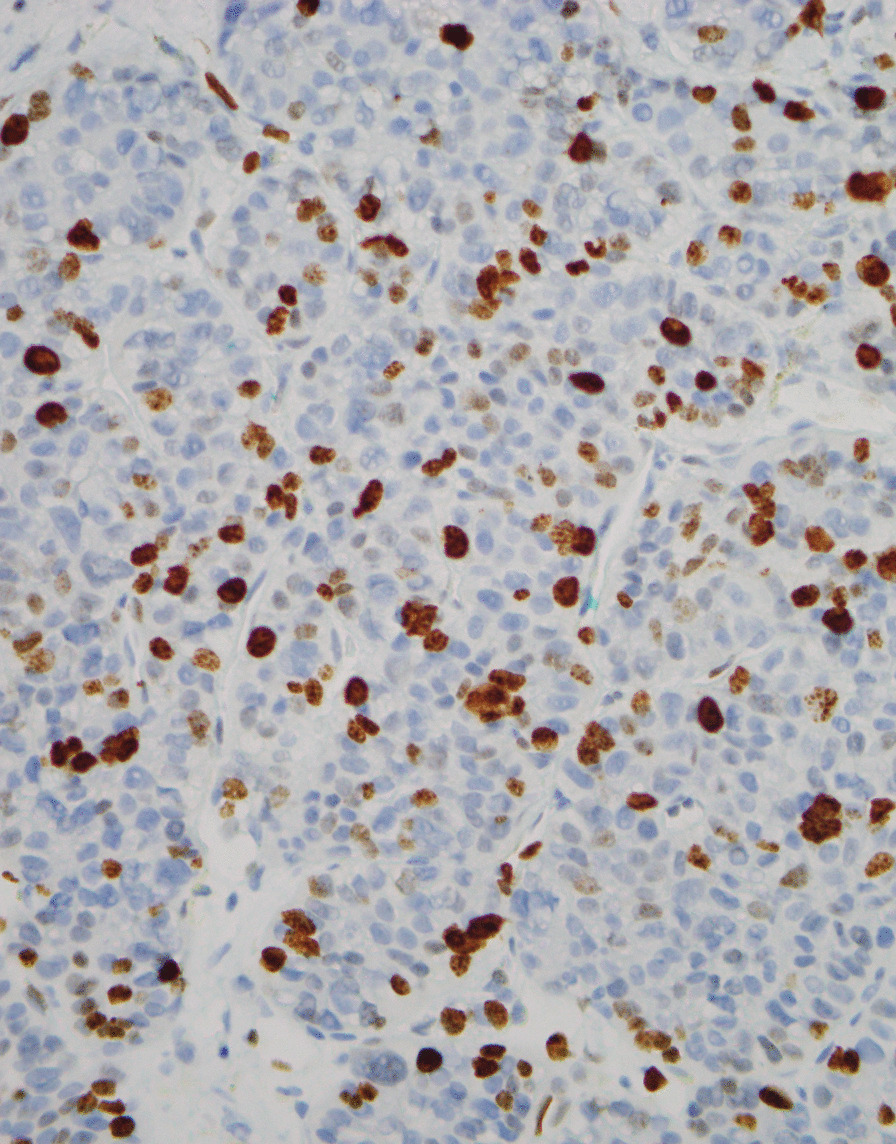
Ki-67 cellular proliferation index 17% tumor cell positivity. Magnification ×400

**Fig. 2:**
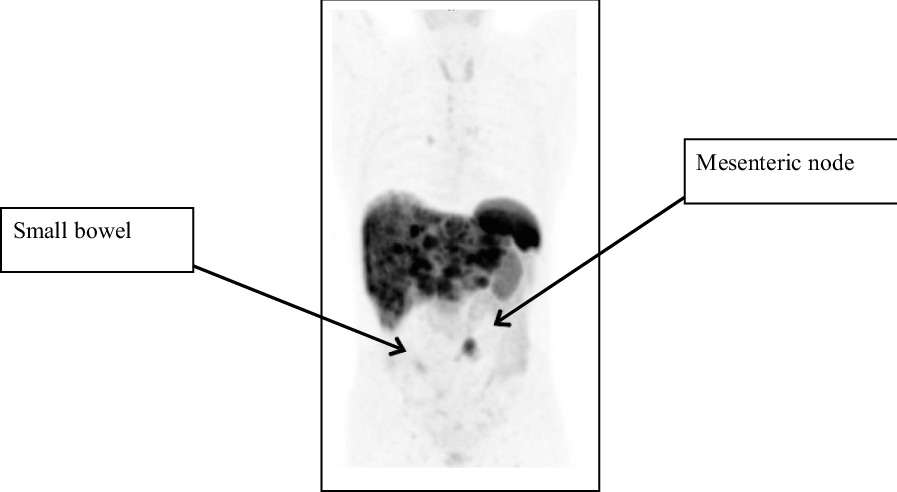
Preoperative ^68^Ga-DOTATATE PET (coronal view)

A previously well 63-year-old Caucasian male presented in 2017 with diarrhea, weight loss, and flushing. Computed tomography (CT) of the abdomen revealed small nonspecific liver lesions, increased in number and size on repeat CT 12 months later. Hepatic core biopsy demonstrated a well-differentiated grade 2 NET, Ki-67 index 17% (Fig. 1). ^68^Ga-DOTATATE position emission tomography (PET) revealed avidity in multiple hepatic lesions and likely small bowel primary (Fig. 2).

The figure shows extensive DOTATATE avid hepatic metastases, diffusely infiltrating both lobes, increased uptake in a mesenteric nodal metastasis, and focal uptake in small bowel, consistent with uptake at the primary site in small bowel.

After undergoing ileocecal resection for control of increasing local symptoms, he developed significant watery diarrhea with up to five bowel motions per day. While it was difficult to obtain a detailed history from the patient, the diarrhea appeared to deteriorate rapidly after surgery; hence, “short gut syndrome” or bile acid diarrhea were considered. However, given the nature of his symptoms with explosive large-volume stools, fecal urgency, and persistence during periods of fasting, his diarrhea was felt to be the result of carcinoid syndrome. This was supported by an elevated 24-hour urinary 5-hydroxyindoleacetic acid (5-HIAA) (Table 1).

**Table 1:** Summary of relevant investigations for cause of diarrhea

Investigation		Result
Serum hormonal levels	Chromogranin A (< 85 ng/mL) Vasoactive intestinal peptide (VIP) (0.0–50.0 pmol/L) Glucagon (50–150 pg/mL)	29,000 Within range Within range
Urine testing	Urine 5-HIAA (0–40 µmol/24 hours)	1727
Pathology	Full blood count, liver function tests, B12, thyroid function, folate, vitamin D, celiac serology, calcium	No significant abnormalities
Fecal analysis	Culture and sensitivity, calprotectin, chymotrypsin, osmolality, pancreatic elastase	No significant findings
Anatomical defects	Flexible sigmoidoscopy Gastroscopy and colonoscopy, colonic biopsy	No significant findings

Following surgery, he commenced 60 mg long-acting octreotide (Sandostatin) monthly but was hospitalized with ten bowel motions per day requiring intravenous fluid hydration and rigorous electrolyte replacement for correction of hypokalemia. Due to significant hepatic burden, severe symptoms, and concern for oral absorption due to the rapid gut transit time, systemic intravenous chemotherapy (modified FOLFOX6-5-fluorouracil, leucovorin, and oxaliplatin) was given. Despite his high volume of disease, synthetic liver function was preserved. Imaging after six cycles of FOLFOX showed stabilization of disease volume, but diarrhea only transiently improved after each cycle. Numerous medications were trialed concurrently with chemotherapy; short-acting octreotide, pancreatic enzyme supplements, opioids (codeine phosphate), serotonin receptor antagonists (ondansetron), tryptophan hydroxylase inhibitor (telotristat), and interferon-alpha; while the combination of these agents, including interferon-alpha, caused a temporary plateauing of his symptoms, none had a significant sustained effect.

His symptoms repetitively deteriorated on discharge yet plateaued on readmission while continuing the same pharmacological management, questioning community compliance. Multidisciplinary team members including pharmacists and dieticians were involved and assisted patient education, yet his diarrhea remained refractory each time he was discharged from hospital. Dual PET imaging with ^68^Gallium DOTATATE and ^18^F-FDG PET demonstrated persisting extensive DOTATATE avid hepatic metastases with minimal FDG uptake. Given his extensive disease and intractable symptoms ^177^Lu-DOTA-octreotate (LuTate) therapy was undertaken to modify functional status by decreasing hormonal secretion. Interferon-alpha was ceased prior to LuTate owing to concerns it was contributing to malaise and fatigue. CT imaging 8 weeks post cycle 1 revealed radiologically stable disease, but serum liver function tests deteriorated (ALP 650 U/L, AST 150 U/L, ALT 145 U/ L, GGT 1390 U/L, bilirubin 165 μmol/L); further LuTate was deemed unsafe. The patient was maintained on intramuscular octreotide 60 mg/second weekly, intramuscular interferon 3×/week, and a continuous ambulatory delivery device (CADD) of 1200 μg/24 hours octreotide and was discharged to a regional hospital. His condition rapidly deteriorated, and he died 8 weeks later.

## Case presentation 2

A 45-year-old Caucasian lady with a history of a pituitary prolactinoma and parathyroidectomy was diagnosed with multiple endocrine neoplasia type 1 (MEN1), with germline testing positive for menin; no family members were affected. Two years later, CT imaging revealed pancreatic and left adrenal lesions; given her disease was resectable, the surgeon proceeded to operate and she underwent a distal pancreatectomy, splenectomy, and left adrenalectomy, pathology of which demonstrated multifocal pancreatic islet cell tumors (largest 40 mm with low-grade features; three mitoses per ten high-power fields, and Ki-67 < 5%). Five years later, she underwent a partial pancreatectomy for local reoccurrence; pathology demonstrated multiple small pancreatic neuroendocrine tumors (largest 10 mm with low-grade features; less than one mitosis per ten high-power fields, and Ki-67 1%).

**Fig. 3:**
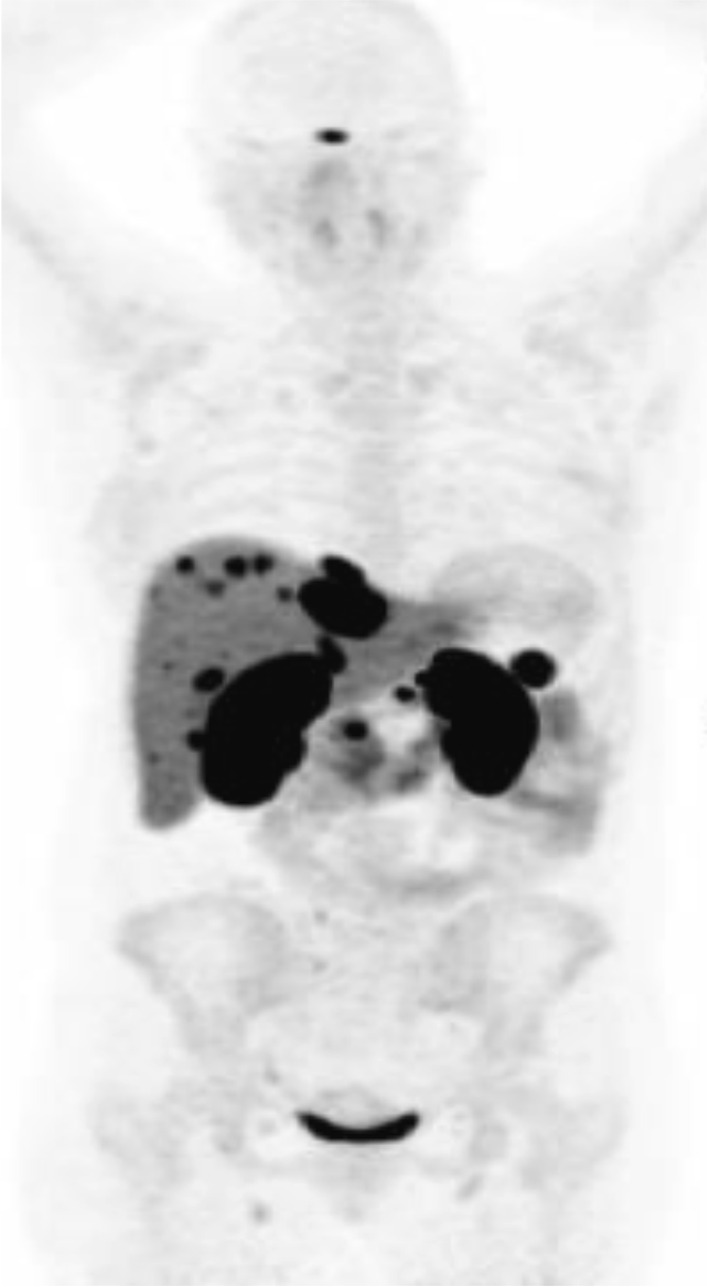
^68^Ga-DOTATATE PET surveillance imaging post-surgery demonstrating multiple liver metastases

The following year, liver metastases were detected on surveillance imaging, and at this time she developed symptoms of odorless, watery diarrhea and flushing (Fig. 3). Extensive investigations were initially unable to explain the etiology of her diarrhea (Table 2).

**Table 2:** Summary of relevant investigations for cause of diarrhea

Investigation		Result
Serum hormonal levels	Vasoactive intestinal peptide (VIP) (0.0–50.0 pmol/L) Glucagon (50–150 pg/mL) Chromogranin A (< 85 ng/mL)	34–initial 408.9–progress 118 19
Urine testing	Urine 5-HIAA (0-40 µmol/24 hours)	21
Pathology	Full blood count, liver function tests, B12, thyroid function, folate, vitamin D, celiac serology, calcium	No significant abnormalities
Fecal analysis	Culture and sensitivity, calprotectin, chymotrypsin	No significant findings
Anatomical defects	Colonoscopy	Only mild colonic inflammation

On repeat hormonal screening, diarrhea was felt to be secondary to VIP hypersecretion from liver metastases [VIP: 408.9 (< 50) pmol/L], and she was commenced on lanreotide 120 g every 4 weeks with some initial improvement in symptoms. Her diarrhea deteriorated despite numerous medications, including codeine, loperamide, diphenoxylate/atropine, Creon, and up-titration of lanreotide to 240 mg/2 weeks, so she was considered for LuTate therapy. Despite two successive normal serum creatinine results, while fasting for her renal diethylenetriamine pentaacetate (DTPA) scan her renal function was markedly decreased, reflecting her inability to maintain hydration while fasting for these scans. She was hospitalized twice with diarrhea at times exceeding 7 L/day; this resulted in severe electrolyte abnormalities with hypokalemia, hypomagnesemia, and hypochlorhydria, and acute renal impairment from hypovolemia. She required intensive care admission given her high risk of cardiac arrhythmia from electrolyte disturbance and risk of hypovolemic shock. Admittedly, she became so accustomed to high-volume diarrhea that her description of its severity was highly disproportionate to its volume, explaining her delayed reporting of symptoms and late presentation to hospital.

**Fig. 4:**
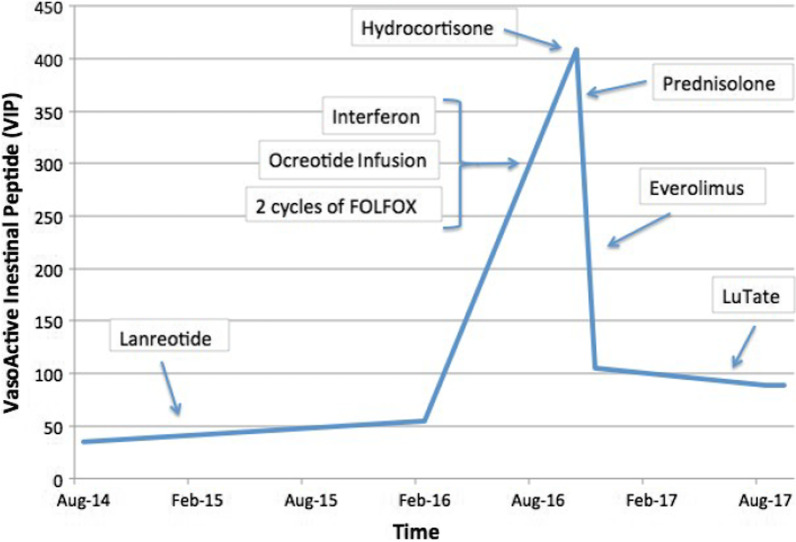
Line graph demonstrating VIP levels against time, with arrows corresponding to the induction of pharmacological interventions

While hospitalized, several anticancer therapies were tried without success, namely two cycles of modified FOLFOX6 chemotherapy, octreotide infusion, and high-dose interferon. None of these agents significantly improved her symptoms. Despite the lack of clinical research into the role of steroids in VIPomas, 100 mg intravenous hydrocortisone was trialed as a last resort. Only on commencement of steroids did her diarrhea frequency and volume (and subsequent biochemical derangement) improve, and she was subsequently discharged on oral prednisolone (Fig. 4). ^68^Ga-DOTATATE PET imaging at this time showed essentially stable dotatate avid disease.

Unfortunately, her diarrhea flared repetitively when steroids were weaned; hence, her course has been complicated by Cushing’s syndrome and steroid-induced diabetes. While her symptoms have fluctuated, recent ^68^Ga DOTATATE imaging has shown radiologically stable disease. She has remained on steroids for 4 years with ongoing labile blood sugar levels. She is planned for review to reattempt to wean the steroids and obtain progress imaging in 6 months.

## Discussion

The two cases demonstrate the heterogeneous nature of neuroendocrine tumors, their ability to secrete different hormones, and their potential to cause chronic, severely disabling, and life-threatening diarrhea. While NETs are rare, the prevalence of diarrhea in NET patients is high, and as this report highlights its potentially fatal nature, guidelines for management and suggestions for novel agents with advancing medicine are essential.

Case 1’s pattern of diarrhea and investigation findings favored a diagnosis of carcinoid syndrome. Given the recurrent improvement in symptoms when hospitalized, and deterioration on discharge, the lack of sustained symptom control and progression of disease was considered at least partially attributable to poor community compliance. This case demonstrates the importance of patient education on the chronic nature of disease and necessity of a multidisciplinary team (MDT) approach to optimize medication compliance to reduce disease morbidity and mortality.

Case 2 suffered high-output odorless, watery, secretory diarrhea that fit the clinical presentation of a VIPoma, in which over 70% of patients experience diarrhea volumes exceeding 3 L/day [3]. Standard management of diarrhea in VIPomas includes antidiarrheal pharmaceuticals, somatostatin analogs, tryptophan hydroxylase inhibitors, surgical tumor debulking, and antineoplastic agents. This case demonstrates that even these extensive treatment modalities may be insufficient for symptom control and is unique in recommending steroids as a novel agent, which proved to have a significant and sustained effect.

While discussing treatment options, it should be noted that both patients underwent early resection of the primary, which is not always first-line management for NETs. There are some retrospective data to suggest a potential survival benefit from primary resection in small bowel NETs [4]. Specific criteria, including the rate of growth, grade, and Ki-67 expression, should be used to guide risk of over-treatment with nonessential resection and the potential complications, against the risk of under-treatment and potential to cure a less aggressive tumor.

We want to emphasize with this report that taking a detailed history regarding diarrhea is imperative as it can be life-threatening. Patients must be educated on the potential danger of severe diarrhea, and to objectively record their symptoms where possible. Case 2 was hospitalized with severe dehydration, hypovolemic renal impairment, and severe electrolyte derangement, and admitted her delayed presentation from under-reporting of symptoms contributed to the severity of her illness. In retrospect clarifying the timeline and severity of symptoms for both patients was challenging, likely due to the gradual insidious onset of NETs. This difficulty in timing the onset of symptoms and diagnosing NET is underlined by the fact that the average time from symptom onset to diagnosis is 52 months [5].

## Conclusion

This report demonstrates two life-threatening cases of diarrhea in NETs, which occurred despite the advent of new systemic therapies. It demonstrates how the key to successful management of diarrhea in NETs requires a close working relationship between the patient and the multidisciplinary team.

### Learning points

- NETs typically have an indolent course, and diarrhea is usually mild and benign. However, these tumors have the potential to cause extremely high-volume diarrhea resulting in severe dehydration, electrolyte disturbance, and renal impairment.
- While carcinoid syndrome is a known cause of diarrhea in NETs, differential diagnoses are broad, and it is imperative to identify the specific pathophysiological causes through thorough history taking and rigorous investigations to guide appropriate treatment modalities.
- The slowly progressive nature of symptoms allows for late and under-reporting by patients, which permits a poor prognostic outcome.
- Management must include extensive patient education and objectively recording symptoms where possible.
- Finally, we suggest a novel option for the management of diarrhea in VIPomas with steroids. While research into glucocorticoid influence on VIP and other peptide hormone production is sparse, this case demonstrates that it warrants further research as it may provide a life-saving treatment in other oncology patients.

## Data Availability

Not applicable.

## Abbreviations

ALP: Alkaline phosphatase
ALT: Alanine aminotransferase
AST: Aspartate aminotransferase
CADD: Continuous ambulatory delivery device
CT: Computed tomography
FDG: (18)F-fluorodeoxyglucose
FOLFOX6: (Modified) 5-fluorouracil, leucovorin and oxaliplatin
GGT: Gamma-glutamyl transferase
LuTate: ^177^Lutetium-DOTA-octreotate
MEN1: Multiple endocrine neoplasia type 1
NETs: Neuroendocrine tumors
PET: Positron emission tomography
VIPoma: Vasoactive intestinal peptide tumor
